# Structure of a short-chain dehydrogenase/reductase (SDR) within a genomic island from a clinical strain of *Acinetobacter baumannii*

**DOI:** 10.1107/S2053230X14019785

**Published:** 2014-09-25

**Authors:** Bhumika S. Shah, Sasha G. Tetu, Stephen J. Harrop, Ian T. Paulsen, Bridget C. Mabbutt

**Affiliations:** a Department of Chemistry and Biomolecular Sciences, Macquarie University, Research Park Drive, Sydney, NSW 2109, Australia; b School of Physics, University of New South Wales, Sydney, NSW 2052, Australia

**Keywords:** opportunistic pathogen, *Acinetobacter baumannii* WM99c, nosocomial strain, multidrug resistance, Rossmann fold

## Abstract

Over 15% of the genome of an Australian clinical isolate of *Acinetobacter baumannii* occurs within genomic islands. An uncharacterized protein encoded within one island feature common to this and other International Clone II strains has been studied by X-ray crystallography. The 2.4 Å resolution structure of SDR-WM99c reveals it to be a new member of the classical short-chain dehydrogenase/reductase (SDR) superfamily. The enzyme contains a nucleotide-binding domain and, like many other SDRs, is tetrameric in form. The active site contains a catalytic tetrad (Asn117, Ser146, Tyr159 and Lys163) and water molecules occupying the presumed NADP cofactor-binding pocket. An adjacent cleft is capped by a relatively mobile helical subdomain, which is well positioned to control substrate access.

## Abstract 2

The structure of a short-chain dehydrogenase encoded within genomic islands of *A. baumannii* strains has been solved to 2.4 Å resolution. This classical SDR incorporates a flexible helical subdomain. The NADP-binding site and catalytic side chains are identified.

## 1. Introduction   

The opportunistic pathogen *Acinetobacter baumannii* is a Gram-negative coccobacillus responsible for both community-acquired and nosocomial infections and is of growing concern owing to the recent emergence of multidrug-resistant forms (Towner, 2009 ▶; Visca *et al.*, 2011 ▶). Colonization appears to be phenotypically associated with inherent systems for exopolysaccharide production, pilus formation and iron sequestration (Iwashkiw *et al.*, 2012 ▶; Peleg *et al.*, 2008 ▶), but specific virulence mechanisms remain poorly described (Durante-Mangoni & Zarrilli, 2011 ▶). Full sequencing of several *A. baumannii* isolates (Ramírez *et al.*, 2013 ▶; Farrugia *et al.*, 2013 ▶; Liou *et al.*, 2012 ▶) has recently highlighted an extraordinary genetic plasticity, with genomic islands (GIs) contributing significantly to diversity of strains. These are identifiable as discrete clusters of laterally acquired DNA segments incorporating factors for chromosomal integration, excision or transfer (Farrugia *et al.*, 2013 ▶). GI features (occasionally termed resistance or pathogenicity islands) are well defined contributors to bacterial evolution, disseminating variable genes influencing niche adaptation and virulence, as well as catabolic genes for new metabolic pathways (Juhas *et al.*, 2009 ▶).

A draft genome sequence of the Australian strain *A. baumannii* WM99c, isolated from a hospital patient in Sydney (Valenzuela *et al.*, 2007 ▶), has recently been described (Eijkelkamp *et al.*, 2011 ▶). At the time of sequencing, our multi-way *BLAST* analysis identified 20 GIs, accounting for 16% of the WM99c genome. One island of interest (GI_12, encompassing 11 ORFs) recurs in its entirety across all known *A. baumannii* genomes of the International Clone II (IC-II) lineage, regardless of geographic origin (Daniel Farrugia, personal communication).

**Figure 1:**
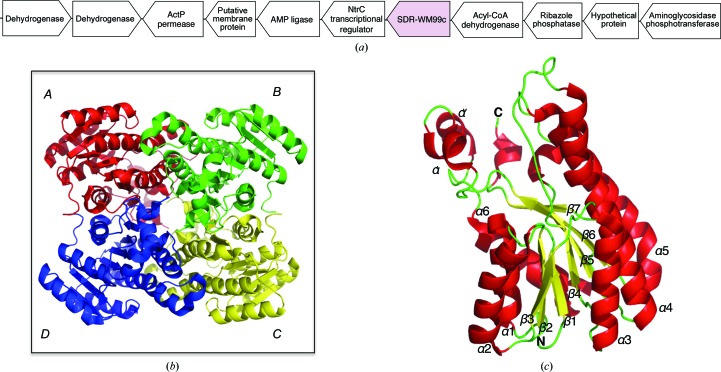
Crystal structure of SDR-WM99c dehydrogenase (apoenzyme form) solved to 2.4 Å resolution (PDB entry 4iuy). (*a*) The genetic organization of the *A. baumannii* GI_12_w_ genomic island encompassing 11 genes. The WM99c gene encoding the SDR-WM99c protein is shaded. Horizontal arrows show the direction of transcription. (*b*) The tetrameric assembly of SDR-WM99c, with chain *A* shown in red, chain *B* in green, chain *C* in yellow and chain *D* in blue, demonstrates two major inter-subunit interfaces (*A*–­*B* and *A*–­*D*). (*c*) A single chain of SDR-WM99c coloured by secondary structure illustrates the three-layered α/β structure with a seven-stranded β-sheet. A helical motif (helices α′ and α′′) caps the C-terminal edge of the sheet and displays some conformational mobility.

The island GI_12 encodes (amongst a group of other enzymes) a permease, a transcriptional regulator and an aminoglycoside phosphotransferase (see Fig. 1 ▶). Here, we describe the 2.4 Å resolution crystal structure determined for SDR-WM99c, one of a pair of hypothetical proteins also encoded within this island. Its structure displays a tertiary fold attributable to a short-chain dehydrogenase (SDR) of classical architecture (Kavanagh *et al.*, 2008 ▶). The relatively large SDR family catalyses a broad range of reactions utilizing NAD(P)-dinucleotide cofactors (Kallberg *et al.*, 2002 ▶). This robust enzyme fold is known to accommodate a wide variety of substrates and thus yields metabolic functions spanning several EC classes from oxidoreductases and lyases to isomerases (Jörnvall *et al.*, 2010 ▶).

This crystal structure defines a new SDR member apparently characteristic to *A. baumannii*. It recurs with identical sequence within GIs from at least 17 distinct strains of the organism, regardless of clonal lineage. Some sequence relatives for this SDR can be discerned in genomes of *Hydrocarboniphaga effusa* (sequence identity 51%), *Marinobacter* sp. (52%) and *Limnobacter* sp. (47%).

## 2. Materials and methods   

### 2.1. Cloning, expression and purification of SDR-WM99*c*   

The gene for SDR-WM99c was PCR-amplified using genomic DNA extracted from *A. baumannii* strain WM99c (Jon Iredell, Westmead Clinical School, Australia). Ligation-independent cloning into vector pET-15b was accomplished using *Bam*HI and *Nde*I restriction sites for expression of SDR-WM99c with an N-terminal His_6_ tag (see Table 1 ▶). Following transformation, *Escherichia coli* BL21 (DE3) pLysS cells (Sigma–Aldrich) were grown at 37°C in selenomethionine medium (M9 kit, Medicilon, Shanghai, People’s Republic of China) until an optical density at 600 nm (OD_600_) of 1.2–1.3 was reached. Recombinant protein expression was induced with 1 m*M* IPTG and the cells were grown overnight with shaking.

Cells were harvested (5000*g*, 30 min) and resuspended in buffer *A* (50 m*M* HEPES buffer pH 7.5, 500 m*M* NaCl) with 5 m*M* imidazole. Cells were lysed by freeze–thawing and sonication on ice. Following centrifugation (20 000*g*, 30 min), the clarified lysate (30 ml) was loaded onto a pre-packed Ni–NTA column (1 ml; GE Healthcare) equilibrated in buffer *A* with 5 m*M* imidazole operating under a peristaltic pump (20°C). Protein product was eluted by competing adsorbent with buffer *A* with 500 m*M* imidazole. After addition of 1 m*M* EDTA, the collected SDR-WM99c fraction was dialyzed into 50 m*M* HEPES buffer pH 7.5 with 300 m*M* NaCl and frozen. The reducing reagent tris-(2-carboxyethyl)phosphine (TCEP) at 0.5 m*M* and glycerol at 5%(*v*/*v*) were additives in all buffers.

The purity of the recombinant product was verified using SDS–PAGE, which showed a single band of 30 kDa. A native mass of 120 kDa was determined for SDR-WM99c by size-exclusion chromatography (SEC) on a Superdex G200 10/300 column in 50 m*M* HEPES buffer pH 7.5, 300 m*M* NaCl, 5%(*v*/*v*) glycerol.

### 2.2. Crystallization and data collection   

Aliquots (0.5–1 µl) of SDR-WM99c (27 mg ml^−1^) were subjected to a sparse-matrix crystal screen (MCSG-1; Microlytic North America) with a Phoenix robot using the sitting-drop format. Optimization was conducted in hanging-drop format (2 µl) in a 24-well grid with 1:1 and 1:2 (protein:reservoir) conditions (see Table 2 ▶). The best diffracting crystals of SDR-WM99c were obtained at 4°C in 0.14 *M* ammonium sulfate, 0.1 *M* HEPES buffer pH 7.5, 18.65%(*v*/*v*) PEG 3350 (crystal 1) and 0.16 *M* ammonium sulfate, 0.1 *M* HEPES buffer pH 7.5, 20%(*v*/*v*) PEG 3350 (crystal 2). Prior to flash-cooling for data collection, crystals were soaked in mother liquor supplemented with 20%(*v*/*v*) glycerol for 25 min (crystal 1) or 10 min (crystal 2) and cryocooled.

X-ray data were recorded on the MX1 beamline at the Australian Synchrotron (Melbourne) using the *Blu-Ice* software (McPhillips *et al.*, 2002 ▶). Reflections were measured on an ADSC Quantum 210r Detector (ADSC, Poway, USA) at a wavelength of 0.9537 Å (13 000.5 eV). For crystal 1, multi-wavelength anomalous dispersion (MAD) data sets were additionally collected. Full data-collection statistics for both crystal 1 and crystal 2 are given in Table 3 ▶.

### 2.3. Structure determination   

Diffraction data were indexed, integrated and scaled with *XDS* (Kabsch, 2010 ▶). Owing to its low sequence identity with the SDR structure family, as well as the weak anomalous scattering of the crystals, solution of the SDR-WM99c structure required a combination of molecular replacement (MR) and single-wavelength anomalous diffraction (SAD). At a lower X-ray dose of 1 s per image, data collected from crystal 1 proved useful to collect the anomalous signal required for calculation of the initial model. Crystal 2 was exposed to a higher X-ray dose (2 s per image), resulting in a comparatively lower anomalous signal but a higher resolution, redundancy and completeness suitable for further refinement. The anomalous scattering of both crystals was not strong enough for utilization of the MAD method to locate the large number of incorporated Se heavy atoms. The space group was determined to be *P*12_1_1 and the asymmetric unit contained eight protein chains with 46% solvent (Matthews coefficient of 2.3 Å^3^ Da^−1^).

A molecular-replacement substructure was obtained with *Phaser MR* (McCoy, 2007 ▶) from the *CCP*4 suite (Winn *et al.*, 2011 ▶) using the structure of the *Salmonella enterica* putative hexonate dehydrogenase (PDB entry 4g81; 35% sequence identity) as a model. The phases from MR and SAD were combined using *Phaser EP* (SAD with molecular-replacement partial structure; McCoy, 2007 ▶). During this MR/SAD pipeline, 40 Se sites were identified in each asymmetric unit. This was followed by density improvement in *Parrot* (Cowtan, 2010 ▶) to yield a preliminary model with 1934 residues. The final model was obtained after several rounds of refinement using *phenix.refine* (Afonine *et al.*, 2012 ▶) in *PHENIX* (Adams *et al.*, 2010 ▶) and manual model building in *Coot* (Emsley & Cowtan, 2004 ▶). The overall stereochemical quality of the final model was assessed using *MolProbity* (Chen *et al.*, 2010 ▶) and the *ADIT* validation server (http://deposit.pdb.org/validate/). Structural homologues were identified with *DALI* searches (Holm & Rosenström, 2010 ▶) as at March, 2014. The coordinates for the final model have been deposited in the Protein Data Bank (PDB entry 4iuy).

## 3. Results and discussions   

### 3.1. Structure determination   

The structure of SDR-WM99c was refined to 2.4 Å resolution with an *R* factor of 15.6% and an *R*_free_ of 20.4%. The enzyme crystallized in the apo form, with no cofactor or substrate bound in any subunit. The final model contains 531 water molecules in each asymmetric unit. All eight chains can be completely traced from residues 2 to 254 (a total chain length of 255 residues). Electron density for the 19-residue His_6_ purification tag was not visible. In all chains, the sole Ramachandran outlier was Ser146 (ϕ = 170.6–173.5°, ψ = 150.9–152.1°). This specific residue is proposed to be part of the active site and a participant in the proton-relay system (see §[Sec sec3.3]3.3). Comparable distortion of active-site serine residues has been noted previously in cofactor-bound and apo forms of several SDR enzymes (*Mycobacterium marinum*, PDB entry 3r1i, Seattle Structural Genomics Center for Infectious Disease, unpublished work; *Synechococcus elongatus*, PDB entry 4dmm, C. Chen, N. N. Zhuang & K. H. Lee, unpublished work; *Rhodobacter sphaeroides*, PDB entry 1k2w, Philippsen *et al.*, 2005 ▶).

The crystalline packing shows that the eight subunits of each asymmetric unit are organized as two homotetramers: *ABCD* (see Fig. 1 ▶ *b*) and *EFGH*. All eight chains align closely, with an r.m.s.d. of 0.3–0.5 Å (C^α^ atoms). Overall, the tetrameric unit *ABCD* displays lower *B*-factor values (see Table 4 ▶). In solution, the native mass determined for SDR-WM99c was consistent with a stable tetramer, and no dimeric population was evident in elution traces.

### 3.2. SDR-WM99c is a classical SDR enzyme   

The fold determined for SDR-WM99c is illustrated in Fig. 1 ▶. Its central parallel β-sheet (strands β1–β7) is surrounded on each side by two groups of α-helices (α1, α2, α6 and α3, α4, α5). Thus, the overall fold is characteristic of the SDR enzyme superfamily (Kallberg *et al.*, 2002 ▶), incorporating a nucleotide-binding Rossmann fold (Rossmann *et al.*, 1974 ▶). The αβα core is extended in the case of SDR-WM99c by a capping helix–turn–helix feature (helices α′ and α′′; Asn197–Ile213). A small 3_10_-helix at the C-terminus makes contact with this capping element. Helices α′ and α′′ display relatively high *B* factors (ranging from 146 to 203 Å^2^), indicating dynamic mobility in this region. As observed across SDR structures (Filling *et al.*, 2002 ▶), a carbonyl group within helix α4 (here, Asn117) ligates to a solvent molecule of the catalytic relay, thus resulting in a characteristic helical kink.

**Figure 2:**
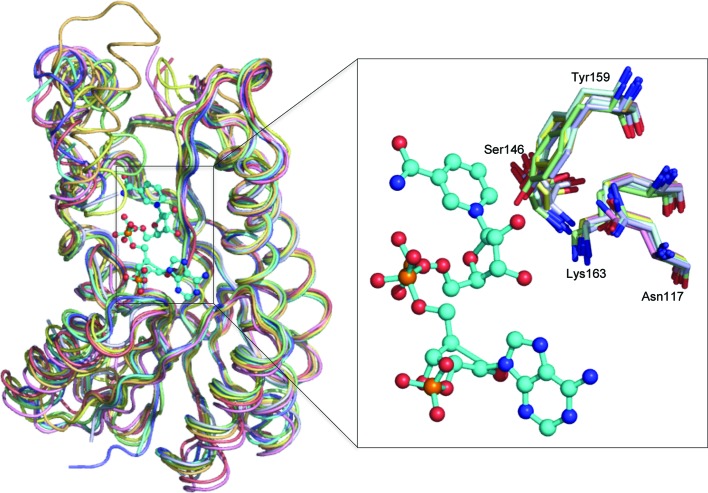
Structure of SDR-WM99c (pink) overlaid with its seven closest structural homologues: 3-oxoacyl-(ACP) reductase (*S. aureus* FabG1; PDB entry 3sj7; cyan), *S. enterica* dehydrogenase (PDB entry 4g81; pale red), 3-oxoacyl-(ACP) reductase (*Synechococcus elongatus* FabG2; PDB entry 4dmm; pale green), 3-oxoacyl-(ACP) reductase (*S. aureus* FabG3; PDB entry 3osu; pale blue), *M. marinum* SDR (PDB entry 3r1i; pale yellow), sorbitol dehydrogenase (*Rhodobacter sphaeroides*; PDB entry 1k2w; pale orange) and oestradiol 17-β-dehydrogenase (PDB entry 2pd6; blue white). The r.m.s.d. is 1.3–1.5 Å over all C^α^ atoms. Inset: magnification of the proposed catalytic side chains Asn117, Ser146, Tyr159 and Lys163 indicates close alignment of the active-site residues. The depicted NADP model is derived from a FabG1 (PDB entry 3sj7) ligand-bound crystal structure.

With the structure of SDR-WM99c solved, a search for structural homologues indicates strong alignment with several members of the classical SDR family. The high degree of structural conservation across this family is seen in Fig. 2 ▶: the closest relatives include an uncharacterized dehydrogenase from *Salmonella enterica* (PDB entry 4g81; Enzyme Function Initiative, unpublished work), FabG1 or 13-oxoacyl-(acyl carrier protein) reductase from *Staphylococcus aureus* (PDB entry 3sj7; Dutta *et al.*, 2012 ▶) and SDH, a sorbitol dehydrogenase from *R. sphaeroides* (PDB entry 1k2w; Philippsen *et al.*, 2005 ▶). FabG1 is involved in fatty-acid synthesis, whereas the SDH enzyme catalyses the dehydrogenation of sugars, with preference for the substrate pair sorbitol/d-fructose. The most marked differences between members of this SDR subgroup arise from variation in the structural arrangement, length and flexibility of the capping helical region (*i.e.* helices α′ and α′′).

### 3.3. Insights into cofactor preference and mechanism of SDR-WM99*c*   

For catalysis, most SDRs depend on a set of four highly conserved active-site features: a serine/threonine, an asparagine and an invariant Tyr-*x*_3_-Lys motif (Filling *et al.*, 2002 ▶). Overlay of SDR-WM99c and its closely related SDR structures (Fig. 2 ▶) immediately reveals retention of the geometry of side chains for this catalytic tetrad. These four active-site residues are thereby identified in SDR-WM99c as Asn117 (α4), Ser146 (β5–α6 loop), Tyr159 (α5) and Lys163 (α5).

**Figure 3:**
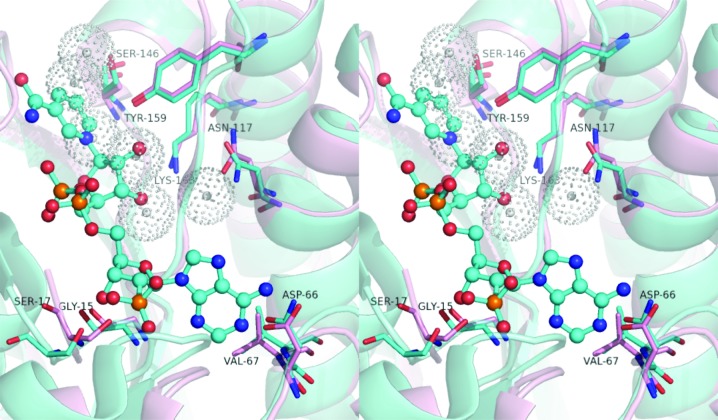
Stereoview of the catalytic site of SDR-WM99c. The active site of SDR-WM99c (pink) modelled with NADP (cyan; superposed from PDB entry 3sj7). Side chains are depicted for active sites from SDR-WM99c (pink) and *S. aureus* FabG1 (cyan; PDB entry 3sj7). Locations of the water molecules across the cofactor site in the crystal structure of SDR-WM99c (apo form) are indicated in grey.

Classical SDR enzymes utilize NADP as a cofactor in catalysis (Filling *et al.*, 2002 ▶). No density consistent with either cofactor or substrate was evident within this structure of SDR-WM99c; however, overlay with the structure of the related FabG1–NADP complex clearly reveals the cofactor site. Fig. 3 ▶ depicts a close view of this region within SDR-WM99c, showing that several water molecules occupy this proposed site for cofactor binding. In the FabG1–NADP structure, the following contacts are documented (Dutta *et al.*, 2012 ▶): the adenine group located near the α4 helix (interacting with Asn60 and Val61), the ribose phosphate surrounded by the β1–α1 loop (Thr8, Gly9 and Arg12) and the nicotinamide moiety stabilized by catalytic residues Tyr152 and Lys156, as well as the β4–α4 loop (Asn87, Ala88). By direct analogy, in SDR-WM99c the adenine ring and the ribose phosphate are proposed to engage α4 helix residues (Asp66 and Val67) and β1–α1 loop residues (Thr14, Gly15 and Ser17), respectively. Additionally, the proposed catalytic residues Tyr159 and Lys163, as well as the β4–α4 loop (Asn93 and Ala94), are well positioned in SDR-WM99c to engage the nicotinamide moiety.

Given the high preservation of the active-site tetrad side chains and nucleotide cofactor-binding pocket, we propose the catalytic chemistry for SDR-WM99c to be as is generally observed across the SDR family (Filling *et al.*, 2002 ▶, Kallberg *et al.*, 2002 ▶; Oppermann *et al.*, 2003 ▶). Thus, the side-chain groups of Tyr159 and Ser146 are proposed to initially hydrogen bond to the substrate. According to the mechanism proposed by Jornvall and coworkers (Filling *et al.*, 2002 ▶), the subsequent proton-relay steps would likely follow: NADP, substrate, Ser146, Tyr159, ribose (cofactor) 2′-hydroxyl group, Lys163, water, Asn117 to bulk solvent. Located in our structure adjacent to Asn117 (2.7 Å) is a water molecule that could be utilized at the end of this proposed relay.

Across the SDR family, a cleft resides close to the cofactor-binding region for substrate binding (Filling *et al.*, 2002 ▶). In SDR-WM99c, such a cleft appears to be located between helices α′ and α′′, *i.e.* in a markedly flexible region of the apoenzyme structure. As the SDR family utilizes diverse chemistry appropriate to a wide range of substrates (Hoffmann & Maser, 2007 ▶), it is not possible to define the specific substrate for SDR-WM99c by homology relationships alone.

The SDR-WM99c enzyme, along with the entire genomic island in which it is encoded, recurs in all sequenced members of the *A. baumannii* IC-II global clonal lineage. Conservation of the island across the IC-II strains suggests that the acquisition of the SDR-WM99c-containing gene cluster by the ancestor of this clonal lineage was a key factor in its success as a globally distributed nosocomial pathogen.

## Figures and Tables

**Table 1:** Macromolecule-production information

Source organism	*A. baumannii* (strain WM99c)
DNA source	*A. baumannii* (strain WM99c)
Forward primer	GCGCGGCAGCCATATGAATATTTTTGATGTAAAAG
Reverse primer	GTTAGCAGCCGGATCCTTATATAGGCGCAGC
Cloning vector	pET-15
Expression vector	pET-15
Expression host	*E. coli* BL21 (DE3) pLysS
Complete amino-acid sequence of the construct produced	MGSSHHHHHHSSGLVPRGSMNIFDVKDKYILITGASSGLGHHIAELFAKEGANIVICARRLERLKELESHIKNEYGVQVYTFALDVNDRSAVKDMLSSLEAEGVTIDVLINNAGVSDTKRFLDYNDEDWDKIVDTNLKAPWQCAQEVVQHMIKAERKGSIINITSILSQSTNLGVSPYCASKAGLRHLTEVMAVELARFGINVNAIAPGYMITEINEEYLTSEVGQQLLKKIPTRKFVEFDDLNGPLLLLASQAGQGITGIEIKVDGGHSAAPI

**Table 2:** Crystallization

	Crystal 1	Crystal 2
Method	Vapour diffusion, sitting drop	Vapour diffusion, sitting drop
Plate type	24-well (Cryschem plate, Hampton Research)	24-well (Cryschem plate, Hampton Research)
Temperature (K)	277	277
Protein concentration (mgml^1^)	27	27
Buffer composition of protein solution	50m*M* HEPES buffer pH 7.5, 300m*M* NaCl, 0.5m*M* TCEP, 5%(*v*/*v*) glycerol	50m*M* HEPES buffer pH 7.5, 300m*M* NaCl, 0.5m*M* TCEP 5%(*v*/*v*) glycerol
Composition of reservoir solution	0.14*M* ammonium sulfate, 0.1*M* HEPES pH 7.5, 18.65%(*v*/*v*) PEG 3350	0.16*M* ammonium sulfate, 0.1*M* HEPES pH 7.5, 20%(*v*/*v*) PEG 3350
Volume and ratio of drop	2l and 1:1 (protein:reservoir)	2l and 1:2 (protein:reservoir)
Volume of reservoir (l)	500	500

**Table 3:** Data-collection and structure-solution statistics Values in parentheses are for the outer shell.

	Crystal 1		Crystal 2
Wavelength ()	0.9537	0.9794	0.9537
Rotation range per image ()	0.5	0.5	0.5
Total rotation range ()	360	360	720
Exposure time per image (s)	1	1	2
Space group	*P*12_1_1	*P*12_1_1	*P*12_1_1
*a*, *b*, *c* ()	106.0, 89.1, 121.1	106.0, 89.1, 121.1	106.2, 89.53, 120.9
, , ()	90, 112.70, 90	90, 112.70, 90	90, 112.69, 90
Resolution ()	19.752.38 (2.512.38)	19.862.45 (2.592.45)	19.762.38 (2.512.38)
No. of unique reflections	80229 (9729)	73908 (9199)	81772 (10613)
Completeness (%)	96.2 (80.5)	96.8 (82.8)	98 (87.8)
Multiplicity	3.7 (2.9)	3.7 (2.9)	7.3 (5.8)
Mean *I*/(*I*)	10.5 (0.9)	10.8 (1.2)	15.6 (1.8)
*R*_merge_ (%)	0.082 (1.078)	0.078 (0.825)	0.087 (0.927)
CC_1/2_	0.997 (0.462)	0.997 (0.541)	0.999 (0.719)
Anomalous completeness (%)	90.4 (61.5)	91.1 (63.9)	93.3 (57.3)
Anomalous multiplicity	1.9 (1.7)	1.9 (1.7)	3.8 (3.6)
CC_anom_	0.174 (0.044)	0.461 (0.013)	0.443 (0.037)

**Table 4:** Structure refinement and model validation Values in parentheses are for the outer shell.

Resolution ()	19.762.38 (2.44502.385)
Cutoff	*F* > 1.34(*F*)
No. of reflections, working set	81728 (4265)
No. of reflections, test set	1993 (91)
Final *R*_cryst_	0.155 (0.2812)
Final *R*_free_	0.203 (0.3959)
No. of protein residues	2025
No. of atoms	
Total	16196
Protein	15665
Solvent	531
TLS groups	32
R.m.s.d. from standard values	
Bond lengths ()	0.008
Bond angles ()	1.15
Average *B* factor (^2^)	
Chain *A* (main chain/side chain)	59.9/65.6
Chain *B* (main chain/side chain)	56.2/63.5
Chain *C* (main chain/side chain)	52.9/59.2
Chain *D* (main chain/side chain)	59.0/67.5
Chain *E* (main chain/side chain)	79.6/85.8
Chain *F* (main chain/side chain)	82.6/88.1
Chain *G* (main chain/side chain)	66.2/73.3
Chain *H* (main chain/side chain)	81.5/88.2
Solvent	57.7
Ramachandran plot (%)	
Favoured regions	97
Allowed regions	2.9
Outliers	0.1
PDB code	4iuy
